# Antenna Excitation Optimization with Deep Learning for Microwave Breast Cancer Hyperthermia

**DOI:** 10.3390/s22176343

**Published:** 2022-08-23

**Authors:** Gulsah Yildiz, Halimcan Yasar, Ibrahim Enes Uslu, Yusuf Demirel, Mehmet Nuri Akinci, Tuba Yilmaz, Ibrahim Akduman

**Affiliations:** 1 Department of Electronics and Communication Engineering, Istanbul Technical University, Istanbul 34469, Turkey; 2 Mitos Medical Technologies, Istanbul 34469, Turkey

**Keywords:** antenna excitation optimization, breast cancer, deep learning, energy focus, microwave hyperthermia, hyperthermia treatment planning

## Abstract

Microwave hyperthermia (MH) requires the effective calibration of antenna excitations for the selective focusing of the microwave energy on the target region, with a nominal effect on the surrounding tissue. To this end, many different antenna calibration methods, such as optimization techniques and look-up tables, have been proposed in the literature. These optimization procedures, however, do not consider the whole nature of the electric field, which is a complex vector field; instead, it is simplified to a real and scalar field component. Furthermore, most of the approaches in the literature are system-specific, limiting the applicability of the proposed methods to specific configurations. In this paper, we propose an antenna excitation optimization scheme applicable to a variety of configurations and present the results of a convolutional neural network (CNN)-based approach for two different configurations. The data set for CNN training is collected by superposing the information obtained from individual antenna elements. The results of the CNN models outperform the look-up table results. The proposed approach is promising, as the phase-only optimization and phase–power-combined optimization show a 27% and 4% lower hotspot-to-target energy ratio, respectively, than the look-up table results for the linear MH applicator. The proposed deep-learning-based optimization technique can be utilized as a protocol to be applied on any MH applicator for the optimization of the antenna excitations, as well as for a comparison of MH applicators.

## 1. Introduction

Microwave breast cancer hyperthermia (MH) is a treatment modality aiming at a non-invasive temperature increase in a malignant breast tumor using electromagnetic (EM) radiation in microwave frequencies [1]. The hyperthermia procedure can be used with other modalities to increase the effectiveness of the cancer therapy [2,3]. Depending on the procedure, a target temperature rise above 39–45 °C is expected [4]. Many MH applicator designs have been proposed [5,6]. After deciding on the applicator, in order to effectively increase the temperature of the malignant tumor and prevent hot spots in the healthy tissue, focusing the microwave energy on the target is essential. Focusing, also called hyperthermia treatment planning (HTP), is achieved through the optimization of the antenna excitations. To this end, many different studies are reported in the literature.

In one of the earlier studies of breast MH, two microwave wave guides were used as the radiation source [7]. During the treatment, Fenn et. al adjusted the excitation phase and amplitude in real-time with a connected phase shifter using the feedback obtained from the invasive measurements of the electric field amplitude and local temperature of the tissue. The time reversal (TR) technique is one of the most used methods in hyperthermia applications due to the general validity of its principle. However, the time-reversal of the energy from the desired target point does not enable regulation of the side lobes. Hence, the technique creates hot spots, especially in lossy media, where TR invariance is no longer valid [8,9,10,11,12,13].

Another proposed approach is to optimize the field distribution in the target region. In [14,15,16,17], the scalar field constrained optimization problem is tackled using convex programming (CP) strategies. For vector fields, the proposed optimization problem becomes a non-deterministic polynomial-time hard (NP-hard) problem, and the authors introduce constrained optimization problem approaches for vector fields in [17,18]. The advantage of constrained focusing over TR is the minimization of the side lobes, since multi-objective optimization minimizes the field outside of the target. These studies show successful results on homogeneous or piece-wise homogeneous scenarios; hot spots are decreased, yet not completely vanished. Due to the nonlinear nature of the problem, the objective function is separated into sub-functions, the optimization is conducted in parallel, and assumptions, such as neglecting the imaginary part of the electric field component, are made.

Nguyen et al. utilized particle swarm optimization (PSO) on 2D and 3D simulation cases, as well as experimental phantom studies [19,20,21]. Antenna array excitations are initially calculated to focus the energy on the target, and then PSO is implemented to adjust the focusing of both energy and temperature distributions with the hybrid utilization of an EM solver and MATLAB. However, the utilized technique has restrictions that prevent its large-scale applicability. For instance, the phase difference is calculated with the distance between antenna arrays, requiring the algorithm to be altered for the application of a different antenna system.

Deep learning has been employed as a tool for solving the medical diagnostics and therapeutics problems in the past decade, such as the registration of multi-modality diagnostic images [22], tissue type classification [23], the optimization of elements in ultrasound thermometry [24], MH monitoring [25], and many more, and it continues to extend its validity. One of the indispensable applications of deep learning is the antenna array selection and excitation optimization problem in beamforming, which is a high-cost problem [26,27,28]. In [28], CNN performs well even in the presence of antenna array imperfections. In [26], an arbitrary radiation pattern was learnt with the use of a deep neural network on antenna excitation phase values.

In this study, a CNN-based approach is proposed for the optimization of antenna excitations in order to enable the focusing of microwave energy in a realistic breast medium. To carry this out, data sets of the possible breast heat distributions and the corresponding antenna excitations using the superposition property are produced. Next, a CNN-based approach is implemented in two steps: first, a CNN is designed to optimize the antenna phases, and, then, in order to optimize the antenna power, the second CNN is designed. In particular, the contributions of this paper can be summarized as follows;

- We propose a CNN-based optimization of the antenna excitation parameters, which can be used as a hyperthermia treatment protocol. The proposed approach is applicable to any MH applicator since it learns directly from the generated dataset. The proposed approach is independent of system parameters such as operation frequency, antenna type, medium, or breast type; therefore, it enables the fair comparison of different MH applicator designs or operation parameters.
- The proposed optimization approach does not depend on the initial value assignment, which may yield a different local best each time it is performed.
- HTP requires multiple cost optimizations and the available optimization techniques solely rely on the given cost function. Combining different cost functions increases the complexity; therefore, most of the techniques do not take these multiple cost functions into consideration. The proposed method does not depend on a cost function, but on a simple mask that substitutes the desired heating map directly.
- We demonstrated the applicability of the proposed CNN-based method with two MH applicator configurations; that is, linear array and circular MH applicators. We used a heterogeneously dense realistic digital breast phantom, which is a difficult breast type to focus the energy. The successful focusing on this breast type demonstrates the capability of the proposed approach.
- CNN models are created offline, but they can be used online for different targets without any time or computational requirements.
- To the best of the author’s knowledge, this is the first paper to utilize deep learning for optimizing the antenna excitations for MH application.
- Finally, this work proposes a fast and simple data generation approach.

The rest of the paper is divided into the following sections: Section 2 lays an overview of the hyperthermia problem, Section 3 gives the details of the followed methodology, Section 4 depicts the results of the proposed approach, along with a comparison to look-up table results, and the conclusions are drawn in Section 5.

## 2. Overview of Hyperthermia Problem

### 2.1. Bio-Heat Equation

The microwave hyperthermia phenomenon is essentially the microwave heat transfer problem within the biological tissues. Penne’s bio-heat equation governs this problem [29]:

(1) $$C_{p}\rho \frac{\partial T}{\partial t}=\nabla \cdot \left(K\nabla T\right)+A_{0}+Q_{0}-B\left(T-T_{b}\right)$$

where $C_{p}$ is the specific heat capacity, ρ is the density, *K* is the thermal conductivity, *T* is the temperature, $T_{b}$ is the blood temperature, $A_{0}$ is the metabolic heat generation, and *B* is the capillary blood perfusion coefficient. These parameters are tissue-specific terms. $Q_{0}$ is the heating potential (HP) and is proportional to the square of the electric field amplitude, and $Q_{0}=0.5\sigma {\left|E\right|}^{2}$ Wm${}^{-3}$, where *E* is the electric field and σ (S/m) is the electrical conductivity. It is shown by Iero et al. [30] that the maxima of HP and the temperature are located at the same position, assuming that *K* and *B* are constants and in a steady-state, via Green’s function approach. Based on the reported work in [30], this paper aims to focus the energy on the target within the HP distribution in order to reach the desired temperature at the target.

### 2.2. Optimization

The total electric field vector inside the breast with N antenna excitations can be written as [31]:

(2) $${\vec{E}}_{tot}\left(r\right)=\sum_{i}^{N}a_{i}{\vec{E}}_{i}\left(r\right)e^{j\phi_{i}}$$

where $\vec{E_{i}}\left(r\right)$ is the electric field vector inside the breast when only the *i*th antenna is excited with unitary excitation, and $a_{i}e^{j\phi_{i}}$ is the *i*th excitation coefficient with $\phi_{i}$ phase difference. The corresponding HP is

(3) $$Q_{0}\left(r\right)=0.5\sigma \left(r\right){\left|{\vec{E}}_{tot}\left(r\right)\right|}^{2}\;W/m^{3}$$

at any location in the breast. The focusing problem requires the maximization of the energy at the target while minimizing it at the healthy tissue; such an objective function Ω can be written as follows [31]:

(4) $$\max_{a_{i},\phi_{i}}\Omega =\frac{\int_{target}\sigma \left(r\right){\left|{\vec{E}}_{tot}\left(r\right)\right|}^{2}}{\int_{breast}\sigma \left(r\right){\left|{\vec{E}}_{tot}\left(r\right)\right|}^{2}}.$$

The electric field is a complex vector with three components in 3D geometry, $|{\vec{E}}_{tot}{\left(r\right)|}^{2}=\left|E_{x}^{2}\left(r\right)+E_{y}^{2}\left(r\right)+E_{z}^{2}\left(r\right)\right|$ and Ex2(r)=(Re(Ex)+jIm(Ex))2. Thus, the optimization problem in (4) is non-linear and a challenging task. In the literature, generally, some assumptions are made, such as Im(Ex)=0 [18], so that the problem is simplified. This work proposes a method to find the optimum antenna excitations without making any assumptions.

## 3. Methods

### 3.1. Antenna Systems and Numerical Test Bed

**Figure 1:**
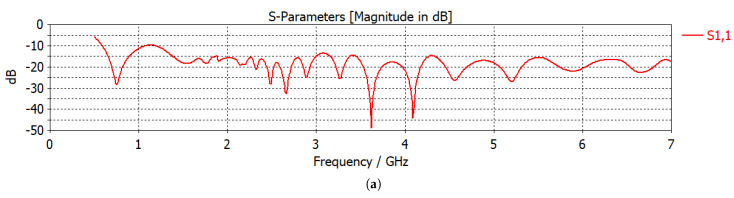

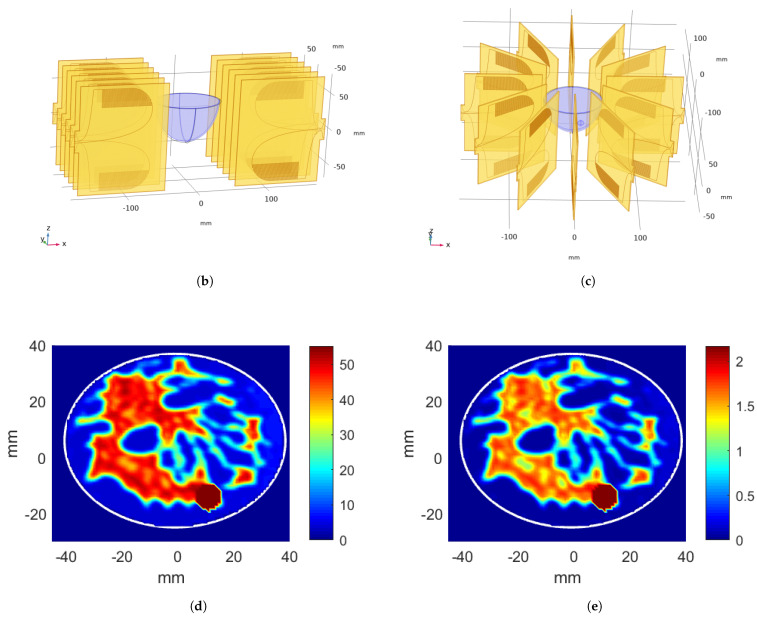
Microwave hyperthermia (MH) applicator configurations: (**a**) S11 parameter vs. frequency graph of a single Vivaldi antenna. (**b**) Linear antenna system, (**b**) circular antenna system with a tumor. Dielectric properties of the central slice of the breast with tumor: (**d**) relative permittivity, (**e**) electrical conductivity (S/m).

Two different antenna configurations, namely hyperthermia applicators, were used in this work: linear and circular. Antipodal Vivaldi antennas, whose frequency range is given in Figure 1a, were used as sources. A 2.45 GHz operation frequency was chosen as it is one of the ISM bands. Figure 1b shows the linear antenna system (linear applicator), where 6 antennas are placed linearly 2 cm apart on one side of the breast and the remaining 6 antennas are placed on the opposite side of the breast. Although there are 12 antennas, we treated 3 successive antennas as an array with same excitation, and N=4 excitations took place for 4 antenna arrays. Consequently, 4 phase and 4 amplitude variables were defined. Please see [6] for a detailed explanation of the antenna configuration and the excitation scheme. Among the 4 phase variables, one of them should be defined as a reference, $\phi_{1}=0$, in order to obtain a unique phase set. From the remaining phase variables, two of them were given a solution space of [0,2π) and, finally, the 4th phase variable was defined as a function of the previous two phase variables, $\phi_{4}=\phi_{2}+\phi_{3}$.

The applicator where the antennas are placed with a circular orientation around the breast tissue is shown in Figure 1c. Antennas were placed into the applicator circularly with 30${}^{\circ }$ angular separation and numbered as shown in the figure. In the circular applicator, there were a total of 12 antennas and the number of antenna excitations was N=12. Even-numbered antennas were allowed to take phase values varying between [0,2π), and the odd-numbered antenna phases were assigned as the summation of two adjacent antennas to lower the degrees of freedom in order to ensure convergence and enable rapid training. Please note that the phase value of 1st antenna was always kept as 0${}^{\circ }$ for reference.

In hyperthermia studies, it is assumed that the patient has been scanned with an imaging modality such as X-ray mammography or magnetic resonance imaging (MRI), and that it is decided that the patient should undergo hyperthermia therapy. It is also assumed that the dielectric properties of the breast is obtained from these imaging modalities. In [32,33], the authors registered the corresponding breast tissues (glandular, fat, skin) to the MRI data and also provided the Cole–Cole and Debye parameters for these tissues. In this study, heterogeneously dense breast with ID 062204 given in [32,33] was used. The dielectric property, namely the relative permittivity ($\epsilon_{r}$) and electrical conductivity (σ) distributions at 2.45 GHz, were calculated using the Debye parameters given in [33]. This is a healthy breast model. Figure 1d,e display the relative permittivity and electrical conductivity from the central slice with a tumor inclusion of the said breast phantom, respectively.

### 3.2. Data Generation

Given the linearity in EM radiation with a single frequency, the superposition property in (2) was used for data generation. One option for data generation is simulation of the whole system with *N* antennas for every potential excitation voltage and phase value. However, both the time and computation cost of said method is high. Therefore, instead, each antenna was excited separately with unitary excitation in a finite element method (FEM)-based multiphysics simulation software (COMSOL Multiphysics) using the *RF module*. While one antenna is excited, remaining N−1 antennas are kept in the system to capture their effect. Electric field ($\vec{E}$) distributions at the central axial slice of the breast, as well as the electrical conductivity σ distributions, were exported for each antenna; that is, a total of N simulations. All possible electric field vectors and heating potential distributions can be constructed using (2) and (3).

The 3D model of the breast and the electrical properties were imported to COMSOL Multiphysics. Antipodal Vivaldi antennas were also imported and positioned as in Figure 1b,c. Note that both the applicator and the said phantom were placed in free space and uniform lumped ports with 50 Ω impedance were used to excite the antennas. During the first set of simulations, for the circular applicator, 1st antenna was excited with 1 volt and 0 phase difference, whereas excitation option of the remaining N−1 ports was chosen as *Off*. The input power was calculated as $P=V^{2}/R={\left(1V\right)}^{2}/\left(50\Omega \right)=0.02$ W. This process was repeated for N=12 excitations. For the linear applicator, on the other hand, all 3 ports of the 3 successive antennas included in the 1st antenna array were excited with 1 volt and 0 phase difference, for the first set of simulations, whereas the ports of the remaining antenna arrays were kept as *Off*. This process was repeated for N=4 excitations. Note that, *Electromagnetic Waves, Frequency Domain* physics, *Frequency Domain* option at single frequency of 2.45 GHz was used throughout this study.

Four cases of applicator and breast were studied: (i) linear applicator and breast without tumor, (ii) linear applicator and breast with tumor, (iii) circular applicator and breast without tumor, and (iv) circular applicator and breast with tumor. During simulations with tumor, a sphere with 5 mm radius was placed on the central slice of the breast phantom. Tumor inclusion had $\epsilon_{r}=40$ and σ=2.0 S/m, which is very close to the maximum electrical conductivity level in the breast [34]. Note that, in the cases (ii) and (iv), it was assumed that the patient was diagnosed with cancer, and that the tumor location, as well as the breast dielectric properties distributions, were known.

**Figure 2:**
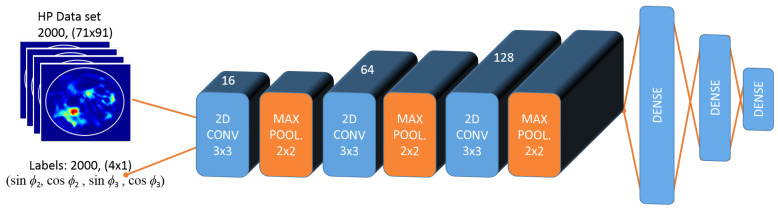
CNN-1 model for the linear system. In order to train the CNN-1 model of the linear system, 2000 data with the size of 71×91 from HP data set was used along with 2000 corresponding labels with the size of 4×1. Labels include the sine and cosine of the two phase values that are optimized in the linear applicator. To utilize the trained CNN-1 model for phase optimization, masked input indicating the desired focus region with a size of 71×91 was given as input, and an estimated 4×1 output was obtained, which has the same structure as the labels.

Data sets for each simulation case were built using (2). The central slice of the breast was utilized; that is, a data set composed from 2D data. First, only phase optimization was considered, in which, the antenna excitation voltages were equal. For the linear system, 2000 $\vec{E}$ distributions were produced with unitary excitations using integer phase pairs that were randomly chosen. Electric fields were then converted to HP distributions using (3). HPs were normalized such that the highest energy level was 1 and lowest energy level was 0. Produced HP data were labeled with the corresponding phase pairs. Note that, instead of using the phase values directly, sine and cosine of the phase were utilized for robustness. As can bee seen in Figure 2, these HP data with size 71 × 91, along with the labels, were used for training the CNN-1, where CNN-1 is the first step of the CNN model fed with the data set, in which, only the phase of the antennas change. CNNs were modeled on Jupyter Notebook using Python’s Keras and TensorFlow modules on an Intel Xeon CPU with 3.10 GHz.

Another data set with 2000 HP distributions was built with optimized phase values obtained from the CNN-1 and varying integer voltage values randomly chosen between 0 and 9. Obtained HP data were then normalized and labeled with the corresponding excitation voltage values. CNN-2, second step of the CNN model, was trained with this data set.

For the circular system, 50,000 normalized HP distributions and corresponding phases were used for training of CNN-1. Using the optimized phase values obtained from CNN-1, another 50,000 HP distributions and corresponding voltages were produced to train CNN-2. Produced data sets were split for training and validation of CNN with 80–20% ratio.

### 3.3. CNN Models

The designed sequential CNN had 3 convolution layers with 3×3 filter size and 16, 64, and 128 filters at respective layers, each followed by a maximum pooling operator of 2×2 as seen in Figure 2. These numbers were selected from common practise examples and adjusted by trial-and-error to meet requirements of this study, which is to build a fast, accurate, and robust optimization scheme. Convolution layers had ReLu activation. Then, batch normalization and flatten layers were applied. CNN model ended with 3 dense layers. The CNN optimizers and parameters were assigned as follows: optimizer was “Adam” optimizer, loss function was “Mean Absolute Error”, batch size was 100 for linear applicator (200 for circular applicator), and learning rate was 0.0001.

**Figure 3:**
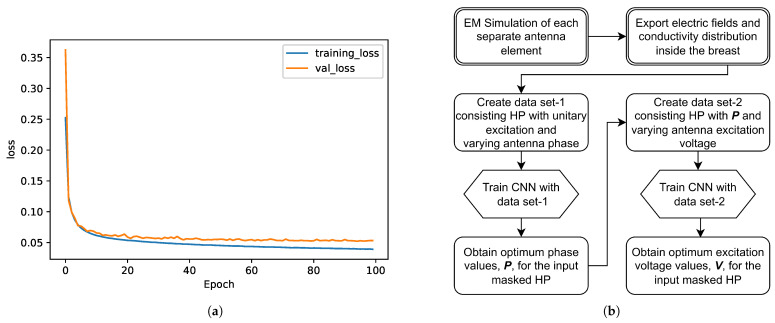
(**a**) Training and validation loss with increasing epoch. (**b**) System flowchart.

The same structure was used for CNN-1 and CNN-2 models. Only difference was the output size of the last dense layer: for linear applicator data set, output size was 4, whereas, for the circular applicator data set, output size was 12. CNNs were trained successfully using the generated data sets. An example of training and validation loss graphic for CNN-2—that is, for circular system voltage level optimization with increasing epoch—is given in Figure 3a. The flowchart for excitation optimization approach is given in Figure 3b.

The exact HP distribution in the breast medium is unknown. To represent the desired HP in the breast medium, a masked HP input was generated. The masked input contains a square region with a 10 mm side length centered at the target point. The target point location coincides with the tumor center if the handled case has a tumor. Since the goal is to maximize the HP on the target and minimize it in the healthy tissue, the value of the square and the remaining region were assigned as 1 and 0, respectively. This masked HP distribution was used as the input to both trained CNN-1 and CNN-2 models, and the outputs were the sine and cosine of the excitation phases for CNN-1 and excitation voltages for CNN-2. Note that the outputs are not necessarily from the data set; since we used CNN for regression, the outputs can take any value within the acceptable range. Obtained excitation voltage levels were then converted to power for convenience.

### 3.4. Evaluation Metrics

The objective function given in (4) was used as an evaluation metric of this study for the discrete data as shown below:

(5) $$\Omega_{target}\left(\Theta ,V\right)=\frac{\sum_{target}\sigma \left(r\right){\left|{\vec{E}}_{tot}\left(r,\Theta ,V\right)\right|}^{2}}{\sum_{breast}\sigma \left(r\right){\left|{\vec{E}}_{tot}\left(r,\Theta ,V\right)\right|}^{2}}*100\%$$

where **Θ** and ***V*** represent the phase and the voltage excitation vectors. Since the tumor radius is 5 mm, the target heating domain was chosen as 10 mm × 10 mm square at the desired location.

In hyperthermia, minimizing the hotspots is almost as important as focusing the energy on the target domain. Since Equation (5) does not account for the hotspots, another metric, named hotspot-to-target ratio, was also utilized in this work. The hotspot-to-target ratio is defined as follows [31]:

(6) $$\Psi \left(\Theta ,V\right)=\frac{\Omega_{hotspot}(\Theta ,V)}{\Omega_{target}\left(\Theta ,V\right)}.$$

In the case of multiple hotspots, the most dominant hotspot was used to evaluate this metric.

Another important parameter is the total input power. In this study, total input power of all of the antennas was fixed to 6 W for all cases, and the results are given accordingly. With the constant total input power of 6 W, the average power deposition at the target domain can be calculated as follows:

(7) $$P_{av}\left(\Theta ,V\right)=\frac{\sum_{target}\sigma \left(r\right){\left|{\vec{E}}_{tot}\left(r,\Theta ,V\right)\right|}^{2}}{\mathrm{target}\mathrm{area}}$$

where the target area was chosen in accordance with the tumor size similar to the earlier target area: 10 mm × 10 mm square at the desired location.

## 4. Results and Discussion

**Figure 4:**
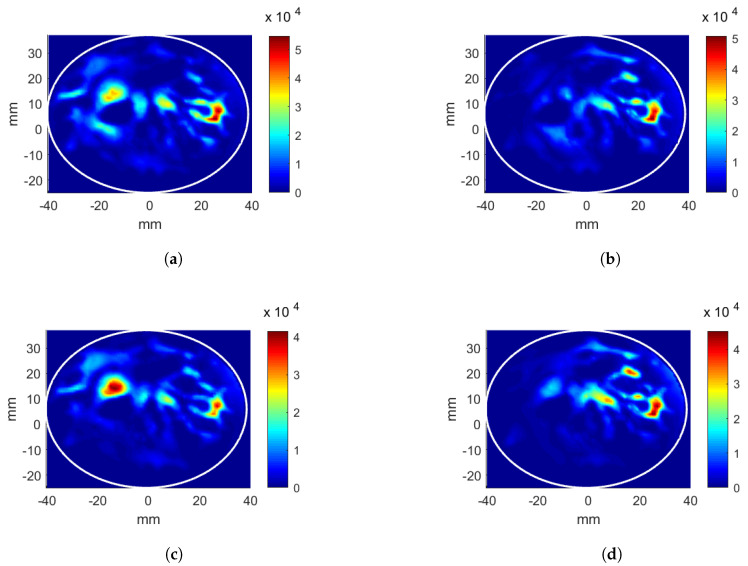
Heating potential (Wm${}^{-3}$) distributions for the linear antenna system and the breast without a tumor, target at (26, 12, 0) mm position. Parameters obtained from CNN: (**a**) phase-only optimization, (**b**) phase and power optimization. Excitation parameters obtained from look-up table: (**c**) phase-only optimization, (**d**) phase and power optimization.

The results of the four applicator and breast cases mentioned previously are given. First, the linear applicator was analyzed with the breast without a tumor inclusion. The position of (x,y,z) = (26, 12, 0) mm was targeted for energy focusing. The CNN-1 model for phase estimation was successfully implemented. The estimated phase values for each antenna array, in order to focus the energy at the target position, are given in Table 1 (shown with A.i). The CNN-2 was trained using these phase values as explained in Section 3.3. The predicted antenna input powers, which were scaled such that the total input power was 6W, are given in Table 1 (shown with A.ii). Note that there are three antennas in each array in the linear applicator, and the input powers are given for each antenna in the corresponding antenna array. The obtained HP distributions after CNN-1 and CNN-2 are given in Figure 4a,b, respectively. Note that the HP distributions are generated with the scaled input power values. The calculated evaluation metric $\Omega_{target}$ is 8.42% after CNN-1 and 10.20% after CNN-2. The hotspot-to-target ratio decreases from Ψ= 1.28 to Ψ= 0.89 points after CNN-2 as $\Omega_{target}$ increases, whereas the $\Omega_{hotspot}$ decreases. $P_{av}$ decreases from 14.89 kWm${}^{-3}$ to 12.01 kWm${}^{-3}$.

In order to evaluate the effectiveness of the CNN-based antenna excitation optimization, a comparison was performed with the results obtained from look-up tables. For the linear antenna system, two phase values ($\phi_{2},\phi_{3}$) were varied to focus the energy on to the target [6]. The HP distributions were calculated for all possible integer phase values (in degrees), and 360 × 360 = 129,600 HP distributions were obtained. For each HP, the evaluation metric of the target, $\Omega_{target}$, was calculated in a simple loop. The phase pair with the highest $\Omega_{target}$ was recorded. Phase values for each antenna element are given in Table 1 (shown with B.i). The obtained $\Omega_{target}$ is 8.69%. The corresponding HP distribution is displayed in Figure 4c. Using the phases obtained from the first look-up table, the antenna voltage excitations vary between 0 V and 1 V, with 0.025 V increments, 41 × 41 × 41 × 41 = 2,825,761 HP distributions were calculated in a simple loop, and the voltage levels that gave the highest $\Omega_{target}$ were recorded. Next, the voltage levels were converted to power and are given in Table 1 (shown with B.ii). The HP distribution obtained from the second look-up table is shown in Figure 4d.

For the phase-only optimizations, the evaluation metric for the target position (x,y,z) = (26, 12, 0) mm obtained using the look-up table is higher than the CNN metric by 0.27%. However, the hotspot-to-target ratio (Ψ) is also higher than the CNN result by 0.63 points, which is not desired in the hyperthermia applications. Since the look-up table is formed by only weighing the evaluation metric at the target location, the hotspots are neglected. On the other hand, well-trained CNN models are known to generalize, and, when the masked input is entered, the CNN model naturally gives the excitations for an HP that is maximized at the target but also minimized at the rest.

An additional power level adjustment improves the evaluation metrics for both CNN and look-up table methods. This second stage not only increases $\Omega_{target}$ but also decreases $\Omega_{hotspot}$, consequently boosting the hotspot-to-target ratio, Ψ, considerably. CNN-2 optimization provides a 1.78% higher $\Omega_{target}$ and 0.39 point lower Ψ than CNN-1; however, $P_{av}$ decreases from 14.89 kW/m${}^{3}$ to 12.01 kW/m${}^{3}$.

**Figure 5:**
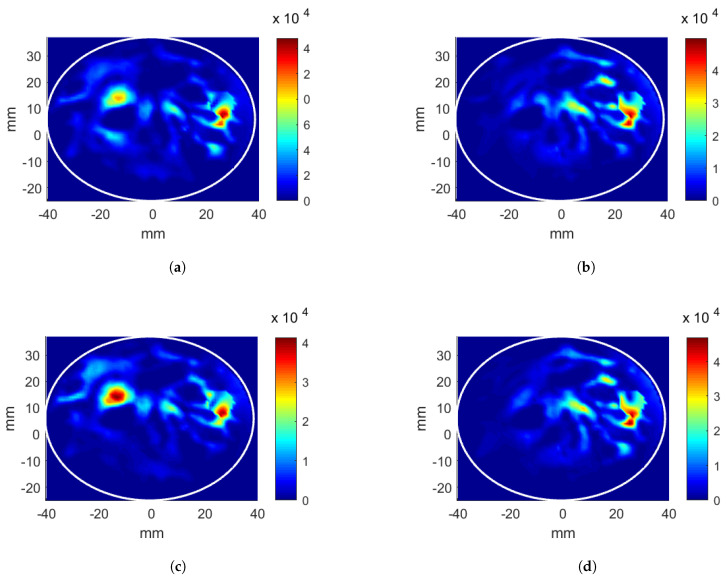
Heating potential (Wm${}^{-3}$) distributions for the linear antenna system and the breast with a tumor at (26, 12, 0) mm position. Excitation parameters obtained from CNN: (**a**) phase-only optimization, (**b**) phase and power optimization. Parameters obtained from look-up table: (**c**) phase-only optimization, (**d**) phase and power optimization.

Second, the linear applicator was analyzed using the breast with a tumor inclusion. The tumor has a 5 mm radius and is positioned at (x,y,z) = (26, 12, 0) mm. The obtained phase values for each antenna array for focusing the energy at the tumor position are given in Table 1 (shown with C.i). The predicted antenna input powers, which are scaled, are shown in Table 1 (shown with C.ii). The HP distributions after CNN-1 and CNN-2 are given in Figure 5a,b, respectively. The calculated evaluation metric $\Omega_{target}\left(P\right)$ is 9.57% after CNN-1 and 12.43% after CNN-2. The hotspot-to-target ratio decreases from Ψ= 0.91 to Ψ= 0.68 points in the second stage as $\Omega_{target}$ increases, whereas the $\Omega_{hotspot}$ decreases. Two-stage CNN optimization provides a 2.57% higher $\Omega_{target}$ and 0.25 point lower Ψ than the first stage; however, $P_{av}$ decreases from 22.72 kW/m${}^{3}$ to 19.55 kW/m${}^{3}$.

The phase values obtained from the look-up table for each antenna array are given in Table 1 (shown with D.i) with corresponding $\Omega_{target}=$ 13.67%. The HP distributions are displayed in Figure 5c,d for the first and the second look-up tables, respectively. For the phase only optimizations, the evaluation metric of the target position (x,y,z) = (26, 12, 0) mm for the look-up table is higher than the CNN-1 metric by 1.04%; however, the hotspot-to-target ratio (Ψ) is also higher than that of CNN-1 by 0.25 points.

The comparison of the obtained results from CNN and the look-up table reveals that using CNN models is a more effective method. Specifically, two-stage CNN has a higher $\Omega_{target}$ and $P_{av}$, and lower Ψ than a two-stage look-up table. Regarding computational costs, for the linear antenna system, the training of CNN-1 and CNN-2 models takes ∼2 min. and ∼4 min., respectively, on the central processing unit (CPU) only, whereas the look-up table search lasts ∼1 min. and ∼29 min., respectively, for the phase and power search using simple loops. Note that neither of the methods are adjusted for time optimization.

Metrics for the breast with a tumor are better than the metrics of the breast without a tumor: higher $\Omega_{target}$, lower Ψ, and higher $P_{av}$ values. This is expected, since the tumor region has a higher conductivity, 2 S/m, than the background conductivity, which varies between 1.00 S/m and 1.88 S/m, and the HP is proportional to conductivity according to Equation (3).

Although the linear applicator metrics obtained via CNN and look-up table methods are compared, the look-up table was not implemented for the circular applicator due to the sheer number of variable combinations. Note that 5 independent phase and 12 independent power values were optimized for the circular applicator. Look-up tables for phase and power require $360^{5}\approx 6$ × $10^{12}$ and $41^{12}\approx 2.25$ × $10^{19}$ loops, respectively. Since the CNN outperformed the look-up table in both evaluation metrics, $\Omega_{target}$ and Ψ, for the linear antenna system, only the CNN method was chosen to optimize the circular applicator parameters.

CNN-1 and CNN-2 models were created for the case with the circular applicator and the breast phantom without a tumor. The parameters obtained from the two CNNs for a target position (x,y,z)= (7, −11, 0) mm are given in Table 2 (shown with A.). The evaluation metrics are calculated as $\Omega_{target}=$ 14.31%, $\Omega_{hotspot}=$ 7.81%, and Ψ= 0.55 for CNN-1, and $\Omega_{target}=$ 15.42%, $\Omega_{hotspot}=$ 7.79%, and Ψ= 0.51 for CNN-2. CNN-2 has a 1.11% higher $\Omega_{target}$ than phase-only optimization and a 0.04 points lower hotspot-to-target ratio. The $P_{av}$ increased from 13.10 kW/m${}^{3}$ to 18.09 kW/m${}^{3}$ with CNN-2.

The parameters obtained for another target position (−11, −7, 0) mm are given in Table 2. (shown with B.). The evaluation metrics for CNN-1 are: $\Omega_{target}=$ 12.42%, $\Omega_{hotspot}=$ 7.95%, and Ψ= 0.64. The evaluation metrics for CNN-2 are: $\Omega_{target}=$ 15.07%, $\Omega_{hotspot}=$ 4.87%, and Ψ= 0.32. $P_{av}$ increased from 10.99 kW/m${}^{3}$ to 12.30 kW/m${}^{3}$ with the second stage of CNN.

**Figure 6:**
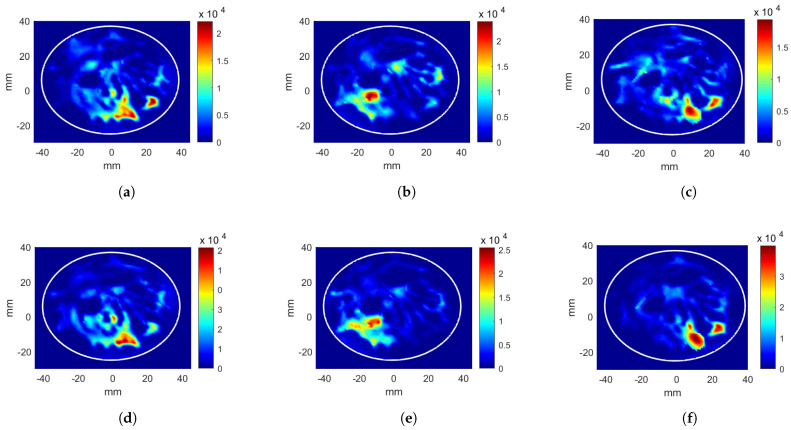
Heating potential distributions (Wm${}^{-3}$) for circular antenna system with excitation parameters obtained from CNN. Phase-only optimization of (**a**) breast without a tumor, target at (7,−11,0) mm position, (**b**) breast without a tumor, target at (−11,−7,0) mm position, and (**c**) breast with a tumor at (11,−14,0) mm position. Phase and power optimization of (**d**) breast without a tumor, target at (7,−11,0) mm position, (**e**) breast without a tumor, target at (−11,−7,0) mm positionm and (**f**) breast with a tumor at (11,−14,0) mm position.

The circular applicator with the breast containing a tumor at (x,y,z) = (11, −14, 0) mm position is considered. The tumor location is given as the target to CNN models. The phase values and the scaled antenna input powers acquired from CNN-1 and CNN-2 are given in Table 2. (shown with C.). The evaluation metrics are calculated as $\Omega_{target}=$ 12.31%, $\Omega_{hotspot}=$ 7.47%, and Ψ= 0.61 for CNN-1, and $\Omega_{target}=$ 19.47%, $\Omega_{hotspot}=$ 10.51%, and Ψ= 0.54 for CNN-2. After the additional excitation voltage optimization stage, the hotspot-to-target ratio decreased by 0.7 points and $P_{av}$ increased from 9.76 kW/m${}^{3}$ to 22.83 kW/m${}^{3}$. The resulting HPs are given in Figure 6c,f for phase-only and phase and voltage optimizations, respectively.

In [35], five hyperthermia treatment planning optimization techniques are compared to each other in terms of capabilities. The authors concluded that PSO and genetic algorithm (GA) or differential evaluation (DE) [36] optimization techniques are superior to Nelder–Mead simplex [37] and pattern search algorithms and TR in terms of a lower hotspot-to-target SAR ratio, and, further, DE is superior to PSO in terms of the target-to-breast SAR ratio and the average power deposition in the target region. PSO and DE algorithms are dependent on the initial values and are prone to finding a local best solution, as well as the issue of obtaining a different solution each time the techniques are used. Antenna excitation parameter optimization is a multi-objective optimization, and the higher the antenna number, the more complex the optimization becomes. Moreover, these optimization techniques are dependent on the cost function of choice, and HTP requires multiple cost functions to be optimized at the same time. The proposed method, on the other hand, presents a simpler solution, with the usage of a simple mask representing the desired HP distribution. A comparison of the mentioned techniques and the proposed method will be conducted in more detail in a later study. Another future work would consist of a 3D optimization study, and simultaneous phase and power optimization.

## 5. Conclusions

In this paper, we propose an alternative approach for finding the optimum antenna excitations for energy focusing in microwave breast hyperthermia. Optimization algorithms proposed so far on this subject generally deal with linear and real functions. On the other hand, the optimization of non-linear and complex functions, such as heating potential, requires mathematical simplifications to ensure convergence. There is a need for a method to tackle the problem completely, without assumptions. Moreover, there is a need for a protocol of extracting the optimum antenna excitation parameters, also known as the input power and the phase, for any MH system with an arbitrary applicator, matching medium, etc. To this end, we exploited the learning ability of convolutional neural networks and let CNN models learn the best fit for any MH system. Linear and circular MH applicators were used to show the generality of the proposed method, along with healthy and tumorous numeric breast phantoms. A heterogeneously dense breast phantom was used as a challenge. Data sets for phase and power optimization were created offline for training, validation, and testing. A step input data highlighting the desired focusing region was utilized for online optimization. Based on the comparison of the proposed method and look-up table results on the linear MH applicator, CNN-optimized excitation parameters produce HP distributions with a smaller hotspot-to-target ratio. It was also demonstrated that our approach performs well on the circular applicator, which has a challenging optimization problem due to the high number of variables. 

## Figures and Tables

**Table 1:** Estimated linear antenna excitation parameters for target at (26, 12, 0) mm.

Study	Excitation	Antenna Array No.				$\Omega_{\mathrm{target}}$	$\Omega_{\mathrm{hotspot}}$	Ψ	$P_{\mathrm{av}}$
Case	Parameters	1	2	3	4	%	%		(kWm${}^{-3}$)
A. CNN,	i. Phase (deg)	0.00	−48.46	−178.10	133.44	8.42	10.78	1.28	14.89
without tumor	ii. Power (W)	0.44	0.27	0.10	1.19	10.20	9.04	0.89	12.01
B. Lookup T.	i. Phase (deg)	0.00	−100.00	138.00	38.00	8.69	16.64	1.91	10.67
without tumor	ii. Power (W)	0.20	0.00	0.33	1.47	10.56	12.71	1.20	12.06
C. CNN,	i. Phase (deg)	0.00	−43.10	135.73	92.63	12.63	11.44	0.91	22.72
with tumor	ii. Power (W)	0.15	0.10	0.03	1.72	15.20	10.08	0.66	19.55
D. Lookup T.	i. Phase (deg)	0.00	−100.00	140.00	40.00	13.67	16.05	1.17	17.11
with tumor	ii. Power (W)	0.33	0.0	0.0	1.67	15.10	10.24	0.68	18.62

**Table 2:** Estimated circular antenna excitation parameters.

Study	Excitation	Antenna No.											
Case	Parameters (deg, W)	1	2	3	4	5	6	7	8	9	10	11	12
A. CNN	i. Phase	0.00	98.57	−15.76	−114.3	170.8	−74.84	−40.73	34.11	−62.28	−96.39	−108.0	−11.60
without tumor ^1^	ii. Power	0.05	0.28	0.01	0.32	0.15	0.34	1.09	1.22	0.15	0.86	0.76	0.79
B. CNN	i. Phase	0.00	126.03	−61.00	172.97	97.59	−75.38	−94.68	−19.31	−59.18	−39.88	96.33	136.21
without tumor ^2^	ii. Power	0.34	0.15	0.00	0.15	0.90	0.90	0.82	0.20	1.84	0.25	0.46	0.00
C. CNN	i. Phase	0.00	−31.33	138.53	169.86	52.28	−117.6	0.42	118.0	29.80	−88.19	−157.3	−69.07
with tumor ^3^	ii. Power	0.02	0.40	0.11	0.19	0.69	0.02	0.00	0.01	0.39	1.85	1.04	1.26

Targets at: ^1^ (−7, 11, 0) mm, ^2^ (−11, −7, 0) mm, ^3^ (11, −14, 0) mm positions.

## Data Availability

Not applicable.

## Abbreviations

The following abbreviations are used in this manuscript:

MH	Microwave Hyperthermia
SAR	Specific Absorption Rate
CNN	Convolutional Neural Network
EM	Electromagnetic
TR	Time Reversal
CP	Convex Programming
NP	Non-deterministic Polynomial-time
PSO	Particle Swarm Optimization
HP	Heating Potential
ISM	Industrial Scientific Medical
MRI	Magnetic Resonance Imaging
FEM	Finite Element Method
CPU	Central Processing Unit
